# Association of germline variants in the *ZFX* gene with primary hyperparathyroidism

**DOI:** 10.1371/journal.pone.0329388

**Published:** 2025-08-08

**Authors:** Ainhoa Camille Aranaga-Decori, Pedro González, Sara Gómez-Conde, Leire Madariaga, Nuria Valdes, Luis Castaño, Alejandro García-Castaño, for the Calcium and Phosphorus Metabolism Molecular Biology Group

**Affiliations:** 1 Biobizkaia Health Research Institute, Barakaldo, Bizkaia, Spain; 2 University of the Basque Country (UPV/EHU), Leioa, Bizkaia, Spain; 3 Endocrinology and Nutrition Department, Cruces University Hospital, Barakaldo, Bizkaia, Spain; 4 Biomedical Research Network Center for Diabetes and Associated Metabolic Diseases (Centro de Investigación Biomédica en Red de Diabetes y Enfermedades Metabólicas Asociadas, CIBERDEM), Biomedical Research Network Center for Rare Diseases (Centro de Investigación Biomédica en Red de Enfermedades Raras, CIBERER), EndoERN, Barakaldo, Bizkaia, Spain; 5 Pediatric Nephrology Department, Cruces University Hospital, Barakaldo, Bizkaia, Spain; IPATIMUP/i3S, PORTUGAL

## Abstract

Somatic variants in the *ZFX* gene have been found in human sporadic parathyroid adenomas. This gene encodes a transcriptional factor recently described as a transcriptional activator in multiple types of human tumors. We present the clinical and molecular characterization of three patients diagnosed with primary hyperparathyroidism (PHPT) who have germline variants in the *ZFX* gene. The first patient had a pathogenic missense variant (c.2321A > G; p.(Tyr774Cys)) in the heterozygous state. This patient exhibited PHPT along with ear, nose and forehead abnormalities. Additionally, she presented other characteristics seen in patients with pathogenic variants in the *ZFX* gene, such as hearing loss and multiple cutaneous nevi. The second and third patients had a missense variant of uncertain significance (c.1606C > T; p.(Arg536Cys)) and an in-frame insertion (c.452_460dup; p.(Gly151_Val153dup)) of uncertain significance, respectively, both in the heterozygous state. These patients had no hearing loss, cutaneous melanocytic nevi, or bone or facial deformities. *ZFX* may be one of the genes to be analyzed in women affected by PHPT with suspected genetic inheritance, especially if they have other features such as facial deformities, hearing loss, and cutaneous melanocytic nevi.

## Introduction

Primary hyperparathyroidism (PHPT) is a condition characterized by the excessive secretion of parathyroid hormone (PTH) by the parathyroid glands, leading to elevated calcium levels [1]. PHPT can be either sporadic (85–95% of cases) or inherited (5–15% of cases). The inherited form can be part of familial syndromes, including multiple endocrine neoplasia types, hyperparathyroidism-jaw tumor syndrome, familial hypocalciuric hypercalcemia, or familial isolated hyperparathyroidism. Additionally, somatic or germline pathogenic variants of the genes responsible for heritable forms also contribute to sporadic PHPT [2].

Soong CP *et al*. found somatic variants in the *ZFX* gene (Zinc Finger Protein X-Linked, MIM 314980) in human sporadic parathyroid adenomas [3]. This gene is mapped to chromosome Xp22.11 [4] and encodes the Zinc finger X-chromosomal protein, which belong to the Krueppel C2H2-type zinc-finger protein family. This protein has 805 amino acids divided in three domains: a nuclear localization sequence, an acidic transcriptional activation domain, and a DNA binding domain consisting of 13 C2H2-type zinc fingers [5], the final three being necessary and sufficient for recruitment to CpG island promoter regions [6,7]. These somatic variants identified by Soong CP *et al*. are located in the zinc finger type 13 domain near the C-terminus of the protein (p.(Arg786Gln), p.(Arg786Leu) and p.(Arg787Thr)). The authors suggest that the zinc finger type 13 interacts directly with DNA, and the substitution of a positively charged arginine with a non-charged amino acid could result in abnormal DNA affinity or may be involved in inter-molecular interactions.

The *ZFX* gene has been described as a transcriptional activator [7,8]. Galan-Caridad JM *et al*. conducted studies in mouse embryonic and adult hematopoietic stem cells and showed that this zinc-finger protein is required as a transcriptional regulator for the self-renewal of both stem cell types [8]. Rhie SK *et al*. recently described the ZFX protein as a transcriptional activator in multiple types of human tumors (kidney, colon, prostate, and breast cancer) [7]. Previously, high expression of the ZFX transcription factor had been correlated with proliferation, tumorigenesis, and poor survival of cancer patients [9–11].

Recently, Shepherdson JL *et al*. described germline variants in the *ZFX* gene associated with developmental delay, behavioral abnormalities, hypotonia, and congenital anomalies with a recurrent facial gestalt [12]. Additionally, the authors observed PHPT in four families with missense variants in this gene [12].

In this study, we report three germline variants identified in the *ZFX* gene, which are suspected to be the cause of PHPT.

## Materials and methods

### Ethics statement

The study was approved by the Ethics Committee for Clinical Research of Euskadi (CEIC-E, code E20/31). Patients and their participating relatives provided written informed consent for the genetic study. The research was conducted in accordance with the Declaration of Helsinki on human experimentation, as outlined by the World Medical Association.

### Patients

Three index cases were included in this study: two with familial PHPT and one patient with PHPT diagnosed before the age of 40. The clinical and biochemical characteristics are shown in Table 1. The patients were referred to our laboratory between 1/07/2020 and 31/12/2024. Clinical diagnoses were made by adult endocrinologists at the Cruces University Hospital in Barakaldo, Bizkaia, Spain. Molecular analysis was performed at the Molecular Genetic Laboratory at the Biobizkaia Health Research Institute in Barakaldo, Bizkaia, Spain.

**Table 1:** Laboratory findings of index cases with a genetic variant in the *ZFX* gene.

Patient	Diagnosis	Gender (Female/Male)	Age (years)	Total Serum Calcium (mg/dL)	Serum Phosphate (mg/dL)		25-OH Vitamin D3 (ng/mL)	PTH (pg/mL)	UCa (mg/24h)	Clinical findings	Medical history	Surgical Treatment	Pathology	Recurrence
ME0204	PHPT	F	29	11.8		1.8	7	452	266	Asthenia, abdominal swelling	Hashimoto’s thyroiditis, multiple cutaneous nevi, nephrolithiasis, face abnormalities, hearing loss	Subtotal parathyroidectomy	Parathyroid hyperplasia	YES
ME0607	PHPT	F	54	11.4		3.8	35	80	196	Asymptomatic	Hashimoto’s thyroiditis, osteopenia, hypercholesterolemia	Left upper selective parathyroidectomy	Parathyroid adenoma	NO
CA0185	PHPT	F	36	10.7		3.1	17.8	113	301	Nephrocalcinosis	Myopia	–	–	–

Abbreviations: PTH, Parathyroid Hormone; U, urinary. Reference ranges: Total Serum Calcium [8.1–10.4 mg/dL]; Serum Phosphate [2.5–4.7 mg/dL]; 25-OH Vitamin D3 [25–80 ng/mL]; intact PTH [10–75 pg/mL]; U.Ca [adult woman < 250 mg/24h]; -; not available.

### DNA analysis

Genomic DNA from patients and relatives was extracted from peripheral blood leukocytes using the MagPurix Blood DNA Extraction Kit (Zinexts Life Science Corp., New Taipei City, Taiwan, R.O.C.) following the manufacturer’s instructions. DNA purity and concentration were assessed using the NanoDrop® ND-1000 spectrophotometer (Thermo Fisher Scientific, Waltham, Massachusetts, USA). For Next-Generation Sequencing (NGS), a targeted panel was designed using the Ion AmpliSeq Designer tool (Thermo Fisher Scientific). This panel included the exon regions and flanking intronic sequences of a selection of genes identified as potentially associated with PHPT and/or hypercalcemia (Table 2), based on information from PubMed (https://pubmed.ncbi.nlm.nih.gov), the Human Gene Mutation Database (HGMD, https://www.hgmd.cf.ac.uk), and Online Mendelian Inheritance in Man (https://www.omim.org). Library preparation was performed using the Ion Ampliseq Library Kit v2.0 (Thermo Fisher Scientific) according to the manufacturer’s instructions. Samples were sequenced using the Ion GeneStudio S5 System (Thermo Fisher Scientific). Base calling, read filtering, alignment to the reference human genome GRCh38.p14, and variant calling were carried out using Ion Torrent Suite and Ion Reporter Software (Thermo Fisher Scientific). Amplicons with insufficiently coverage (<20x) and candidate variants were further assessed by Sanger sequencing after polymerase chain reaction (PCR), using fluorescent dideoxynucleotides (BigDye Terminator v3.1 Cycle Sequencing Kit, Life Technologies, Grand Island, NY, USA), and loaded onto an ABI3130xl Genetic Analyzer (Thermo Fisher Scientific). DNA variants were named according to the Human Genome Variation Society guidelines (www.hgvs.org) and classified following the ACMG-AMP (American College of Medical Genetics and Genomics and the Association for Molecular Pathology) guidelines [13].

**Table 2:** Genes associated with PHPT and/or hypercalcemia included in the panel for analysis.

OMIM®	Gene	Phenotype
605314	*HDAC4*	Neurodevelopmental disorder with central hypotonia and dysmorphic facies. PHPT
601199	*CASR*	Hypocalciuric hypercalcemia, type I
139313	*GNA11*	Hypocalciuric hypercalcemia, type II
602242	*AP2*S1	Hypocalciuric hypercalcemia, type III
613733	*MEN1*	Multiple endocrine neoplasia 1
116899	*CDKN1A*	Multiple endocrine neoplasia 1
600778	*CDKN1B*	Multiple endocrine neoplasia, type IV
600431	*CDKN2B*	Multiple endocrine neoplasia 1
603369	*CDKN2C*	Multiple endocrine neoplasia 1
164761	*RET*	Multiple endocrine neoplasia IIA
607393	*CDC73*	Hyperparathyroidism-jaw tumor syndrome
603716	*GCM2*	Hyperparathyroidism 4
168461	*CCND1*	Sporadic PHPT
116806	*CTNNB1*	Sporadic PHPT
601573	*EZH2*	Weaver syndrome, Sporadic PHPT
314980	*ZFX*	Sporadic PHPT
126065	*CYP24A1*	Hypercalcemia, infantile, 1
182309	*SLC34A1*	Hypercalcemia, infantile, 2

## Results

### Clinical and biochemical characteristics

The first patient (ME0204) was a 29-year-old female, who was referred for evaluation of persistent hypercalcemia detected in several routine blood tests. Four years prior, she had been diagnosed with Hashimoto’s thyroiditis, with no other relevant medical history, and had been on levothyroxine treatment since then. She presented with asthenia and persistent hypercalcemia (serum calcium 11.8 mg/dL; reference range 8.1–10.4), low serum phosphate (1.8 mg/dL; reference range 2.5–4.7), elevated intact PTH (452 pg/mL; reference range 10–75), low 25-hydroxyvitamin D levels (7 ng/mL; reference range 25–80), and high serum alkaline phosphatase (117 U/L; reference range 30–106 U/L). The patient showed normal to high urinary calcium excretion (266 mg/24hours; reference range in adult women < 250 mg/24hours), and low tubular reabsorption of phosphate (TRP: 81%), with a maximal tubular reabsorption of phosphate per glomerular filtration rate (TmP/GFR) of 1.9 mg/dL, indicating excessive renal loss of phosphate. Non-obstructive right nephrolithiasis was observed on renal ultrasound. A Sestamibi scan of the parathyroid glands was non-localizing. She underwent bilateral exploration, and all four parathyroid glands were enlarged (parathyroid hyperplasia). Subtotal parathyroidectomy was performed, leaving the upper right parathyroid tissue (upper left parathyroid weight 2.1 g, upper right (partial resection) 1.05 g, lower left 0.25 g, and lower right 2.8 g). She experienced a recurrence of PHPT 5 years after surgery. Currently, at 37 years old, she is taking 60 mg of cinacalcet and has hypercalcemia (serum calcium 11.1 mg/dL) and elevated PTH (302 pg/mL). An F-Choline PET-CT was performed, revealing, in addition to the parathyroid gland left during the previous surgery, two ectopic parathyroid adenomas located in the anterior mediastinum: one at the level of the sternoclavicular notch and the other within the pericardial fat. She also has osteopenia (T-score at the lumbar spine −1.1 SD, T-score at the femoral neck −1.2 SD). Regarding neurological findings, she experiences sleep disturbances. In terms of facial features, she presents ear, nose, and forehead abnormalities, thick eyebrows, and a thin upper lip. She also has hearing loss and multiple cutaneous nevi (melanocytic nevus of the breast, dysplastic nevus of the upper left limb, Spitz nevus of the upper right limb, and intradermal nevus in the groin), fibrous histiocytoma, and hyperhidrosis. Her mother was diagnosed with PHPT at the age of 56 and underwent parathyroid surgery. An upper right parathyroid adenoma was removed (weight 1.17 g). Her last follow-up was at 66 years old, at which point she had hypercalcemia (serum calcium 16.6 mg/dl). She died of sepsis, so a sample for genetic analysis was not available.

The second patient (CA0185) was a 36-year-old female diagnosed with PHPT, with hypercalcemia detected incidentally during the third trimester of her second pregnancy (serum calcium 10.7 mg/dL). She had elevated intact PTH (113 pg/mL), and her 25-hydroxyvitamin D was 17.8 ng/mL. The patient exhibited high urinary calcium excretion (301 mg/24 hours). She also presented with hematuria and severe lower back pain, and renal ultrasound revealed nephrocalcinosis. A sestamibi scan of the parathyroid glands was normal. She is currently evaluating surgery. Phenotypically, she did not have facial deformities or developmental abnormalities, and she had previously undergone surgery for myopia. Her father had a history of multiple episodes of renal colic but does not currently have PHPT. Her mother does not currently have PHPT either.

The third patient (ME0607) was a 54-year-old female diagnosed with PHPT. She had osteopenia (T-score at the lumbar spine −2.2 SD, T-score at the femoral neck −1.9 SD), hypercalcemia (serum calcium of 11.4 mg/dL), and elevated intact PTH (80 pg/mL). Her 25-hydroxyvitamin D level was normal (35 ng/mL). The patient exhibited normal urinary calcium excretion (196 mg/24 hours). She was also diagnosed with Hashimoto’s thyroiditis. A sestamibi scan of the parathyroid glands was compatible with an adenoma located in the left paraesophageal region, posterolateral to the trachea. A left upper selective parathyroidectomy was performed and the pathological examination revealed a 0.3 g adenoma with no atypical features. Four years postoperatively, she maintained normal calcium and PTH levels. Phenotypically, she did not have cutaneous melanocytic nevi, bone or facial deformities, long fingers, scoliosis, or developmental abnormalities. Her mother was diagnosed with PHPT at 64 years old and underwent parathyroid surgery. However, we do not have information about the type of surgery or the pathology. The last follow-up at 88 years old showed no evidence of PHPT.

### Genetic testing

**Fig 1:**
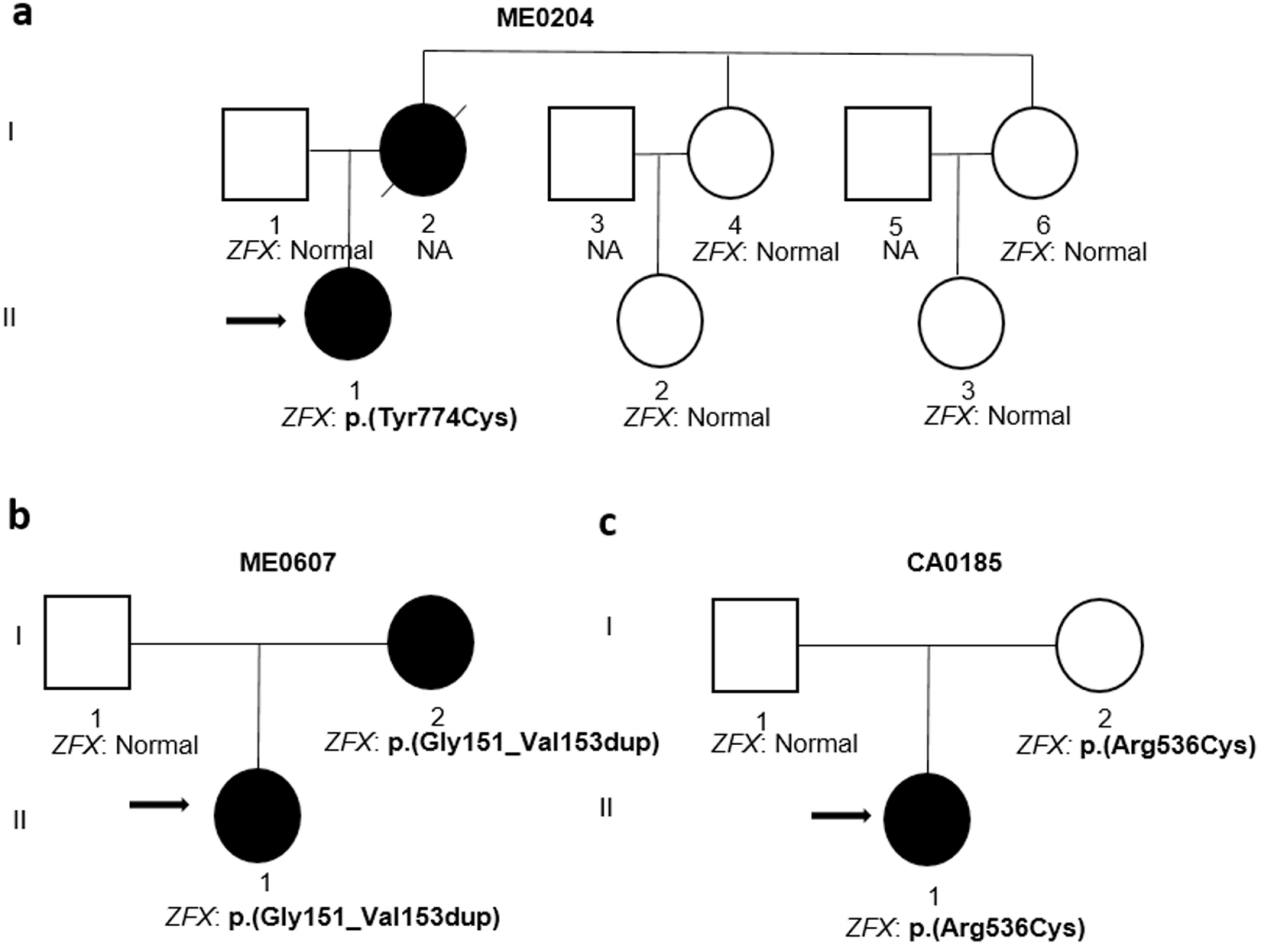
Pedigree of three families with variants in the *ZFX* gene.

Genetic testing identified three variants in the *ZFX* gene (Table 3). Index case ME0204 carried a pathogenic missense variant (c.2321A > G; p.(Tyr774Cys)) in exon 10 (NM_003410.4) in the heterozygous state. The ZFX protein contains 13 C2H2 zinc finger domains (UniProtKB - P17010). This alteration occurs at a highly conserved position between the type 12 and 13 C2H2 zinc finger domains, near the C-terminus of the protein. The p.(Tyr774Cys) variant was not inherited from her father (Fig 1A, I.1). Genetic analysis could not be performed on her mother, who had also PHPT (Fig 1A, I.2). The p.(Tyr774Cys) variant has recently been described as pathogenic [12].

**Table 3:** *ZFX* variants identified in patients.

*Family*	*Number of individuals with the variant*	Nucleotide change*	Amino acid change*	Exon	Domain	Diagnosis	Variant Class	Selected ACMG criteria
ME0204	1	c.2321A > G	p.(Tyr774Cys)	10	DNA binding domain	PHPT	Pathogenic	PS3, PM1, PM2, PP2, PP5
ME0607	2	c.452_460dup	p.(Gly151_Val153dup)	5	Acidic transcriptional activation domain	PHPT	Uncertain significance	BS2, PM2, PM4
CA0185	2	c.1606C > T	p.(Arg536Cys)	10	DNA binding domain	PHPT	Uncertain significance	PM2, PP2

* Numbering is according to DNA sequence (Ensembl: ENST00000304543.10), all in heterozygous; PS, Strong evidence of pathogenicity; PM, Moderate evidence of pathogenicity; PP, Supporting evidence of pathogenicity; BS, Strong evidence of benign impact.

Probands are indicated by the arrows. Squares denote male family members, circles female family members, and solid symbols patients with hyperparathyroidism. NA, not available.

Index case CA0185 carried a missense variant (c.1606C > T; p.(Arg536Cys)) in exon 10 (NM_003410.4) in the heterozygous state. This variant has not been found in the population databases checked (gnomAD, dbSNP). The alteration occurs in the C2H2-type 4 zinc finger (DNA binding domain). The p.(Arg536Cys) variant was inherited from her mother (Fig 1C, I.2), who did not have PHPT.

Index case ME0607 carried an in-frame insertion (c.452_460dup; p.(Gly151_Val153dup)) in exon 5 (NM_003410.4) in the heterozygous state. This variant has a low frequency (Minor allele Frequency < 0.01) in population databases (gnomAD, dbSNP). This duplication occurs in the acidic transcriptional activation domain, near the N-terminus of the protein. The p.(Gly151_Val153dup) variant was inherited from her mother (Fig 1B, I.2), who also had PHPT.

## Discussion

In this study, we described three patients with germline variants in the *ZFX* gene suspected of being responsible for PHPT. Recurrent somatic *ZFX* variants have been identified in human sporadic parathyroid adenomas [3,14,15], and more recently, Shepherdson JL *et al*. described germline variants in the *ZFX* gene associated with PHPT. The variant p.(Tyr774Cys) found in ME0204 is located in a critical region of the ZFX protein, within the linker between zinc fingers 12 and 13 in the DNA binding domain. Shepherdson JL *et al*. demonstrated that this variant affects transactivation ability. They reported that the mutant protein exhibits enhanced binding to DNA in certain promoters, with the majority of differentially regulated genes being upregulated [12]. The authors observed facial gestalt, developmental delay, and hypotonia in four patients with the p.(Tyr774Cys) variant (three male patients in the hemizygous state and one female patient in the heterozygous state). In contrast, among four carrier females, only PHPT was observed [12]. Patient ME0204 exhibited PHPT along with ear, nose and forehead abnormalities, but did not show developmental delay. Additionally, she presented other characteristics seen in patients with pathogenic variants in the *ZFX* gene, such as hearing loss and multiple cutaneous nevi [12,16]. It appears that male patients with pathogenic variants present with the intellectual developmental disorder X-linked syndromic 37 (MIN 301118), while females with heterozygous pathogenic variants may present with the syndromic form, although in the majority of carrier females only PHPT is observed. Further studies are needed to understand why the syndromic form is present in some carrier females.

Index cases ME0607 and CA0185 did not have hearing loss, cutaneous melanocytic nevi, bone or facial deformities, long fingers, scoliosis, or developmental abnormalities. The p.(Gly151_Val153dup) variant found in ME0607 is located in the transactivation domain and has a frequency of 0.0005526 in gnomAD v4.1.0, with an allele count of 669 (number of hemizygotes: 198), which is too high for a dominant pathogenic variant. In the Leiden Open Variation Database (LOVD), it is classified as likely benign with a single entry. On the other hand, this duplication was found in four patients with azoospermia [17] and in one female patient with a developmental disorder [18]. The duplication is inherited from the mother, who also had PHPT. Both remain without recurrence of PHPT after the intervention. The p.(Arg536Cys) variant found in the index case CA0185 is located in the DNA binding domain, and has not been found in the gnomAD population database, nor described in the literature. This variant involves the alteration of a conserved nucleotide. The pathogenicity predictors consulted show discordant results regarding the possible pathogenicity of the variant. This missense variant is located in the Type 4 zinc finger, and, as far as we know, the pathogenic missense variants described so far are located in the final three zinc fingers (Type 11, 12 and 13), which are sufficient for recruitment to CpG island promoter regions [6,7]. The variant was inherited from the mother, who does not currently have PHPT. Therefore, based on the available data, we cannot rule out the possibility that these two variants are involved in a change in the transcription of certain genes. It would be interesting to carry out functional studies to verify the pathogenicity of these variants.

In conclusion, as our data indicate, and other studies have shown, pathogenic variants in the *ZFX* gene can cause PHPT, primarily in carrier females. These patients may present with PHPT, either with or without the syndromic form. Therefore, *ZFX* may be one of the genes to be analyzed in women affected by PHPT with suspected genetic inheritance, especially if they have other features such as facial deformities, hearing loss, and cutaneous melanocytic nevi.
